# Myofilament Calcium Sensitivity: Role in Regulation of *In vivo* Cardiac Contraction and Relaxation

**DOI:** 10.3389/fphys.2016.00562

**Published:** 2016-12-16

**Authors:** Jae-Hoon Chung, Brandon J. Biesiadecki, Mark T. Ziolo, Jonathan P. Davis, Paul M. L. Janssen

**Affiliations:** ^1^Department of Physiology and Cell Biology, The Ohio State University Wexner Medical CenterColumbus, OH, USA; ^2^Dorothy M. Davis Heart and Lung Research Institute, The Ohio State University Wexner Medical CenterColumbus, OH, USA; ^3^Medical Scientist Training Program and Biomedical Sciences Graduate Program, The Ohio State University Wexner Medical CenterColumbus, OH, USA; ^4^Department of Internal Medicine, The Ohio State University Wexner Medical CenterColumbus, OH, USA

**Keywords:** muscle, twitch, kinetics, desensitize, sensitize

## Abstract

Myofilament calcium sensitivity is an often-used indicator of cardiac muscle function, often assessed in disease states such as hypertrophic cardiomyopathy (HCM) and dilated cardiomyopathy (DCM). While assessment of calcium sensitivity provides important insights into the mechanical force-generating capability of a muscle at steady-state, the dynamic behavior of the muscle cannot be sufficiently assessed with a force-pCa curve alone. The equilibrium dissociation constant (K_d_) of the force-pCa curve depends on the ratio of the apparent calcium association rate constant (k_on_) and apparent calcium dissociation rate constant (k_off_) of calcium on TnC and as a stand-alone parameter cannot provide an accurate description of the dynamic contraction and relaxation behavior without the additional quantification of k_on_ or k_off_, or actually measuring dynamic twitch kinetic parameters in an intact muscle. In this review, we examine the effect of length, frequency, and beta-adrenergic stimulation on myofilament calcium sensitivity and dynamic contraction in the myocardium, the effect of membrane permeabilization/mechanical- or chemical skinning on calcium sensitivity, and the dynamic consequences of various myofilament protein mutations with potential implications in contractile and relaxation behavior.

## Introduction

In this review, we discuss the three major mechanisms (Frank-Starling mechanism, heart rate/frequency-dependent contraction, and beta-adrenergic stimulation) that govern cardiac output as well as affect calcium sensitivity, mainly at the level of troponin C, compare calcium sensitivity measurements in skinned/permeabilized and intact muscle preparations, in order to shed light on myofilament protein mutations that have the potential to be translated to further our understanding of cardiac physiology *in vivo*. We recognize that drugs, metabolites, pH, etc., also critically impact on calcium sensitivity (and potentially cardiac output), but that these are typically not primarily resulting from sarcomeric mutations, and were deemed beyond the scope of this review.

At the beginning of the cardiac contraction cycle, calcium ions enter cardiomyocytes via voltage-activated L-type calcium channels, leading to calcium-induced calcium release (CICR) from the sarcoplasmic reticulum (SR). The release of calcium from the SR increases free calcium ion concentration from approximately 100 nM to 1 μM, making more calcium available for binding to troponin C (TnC), a subunit in the troponin complex. Calcium ions binding to TnC initiates a cascade of events leading to force generation via interaction between thin and thick filaments, i.e., by the cycling of cross-bridges. In order to relax, calcium must come off TnC to cease activation, and to allow dissociation of thin and thick filaments to occur and relax the muscle. Calcium ions are recycled back into the SR via SR Ca^2+^ ATPase (SERCA) or extruded out of the cell via Na^+^/Ca^2+^ exchanger (NCX).

Myofilament calcium sensitivity is a concept used by researchers to simplify the complex, dynamic process of cardiac contraction, and relaxation into a relationship between the concentration of free calcium ions available for binding to TnC and the amount of force generated by the muscle. In failing myocardium, calcium sensitivity has been reported to either increase or decrease depending on the etiology of the disease (Willott et al., 2010). As the calcium sensitivity increases, the contractility of the muscle typically increases, but this also means that relaxation may be often impaired if calcium dissociates from TnC more slowly. The vast majority of previous studies have utilized mechanically and chemically skinned muscle preparations for measuring calcium sensitivity because they are able to reduce a complex system into one that only contains two variables: free calcium ions and force of contraction by the myofilaments. This reductionist approach has revealed important mechanical properties of the thin and thick filaments but does not sufficiently translate into the contracting heart. The force-generating capacity of cardiac muscle *in vivo* takes into account not only the simple association and dissociation rate of calcium from TnC but the entire intracellular environment that includes various kinases and phosphatases, for example. From previous studies, it is clear that the myofilaments play an integral role in cardiac muscle contraction and relaxation. Therefore, the myofilaments are an important target in treatment of heart failure, which continues to afflict millions of lives today with limited treatment options. It is imperative that we utilize the invaluable knowledge the cardiac muscle physiology field has already generated regarding calcium sensitivity and produce new data to not only further our understanding of the physiology of a dynamically contracting heart *in vivo* but also more effectively translate our findings to the clinic.

## Calcium sensitivity and dynamic behavior of a muscle

**Figure 1:**
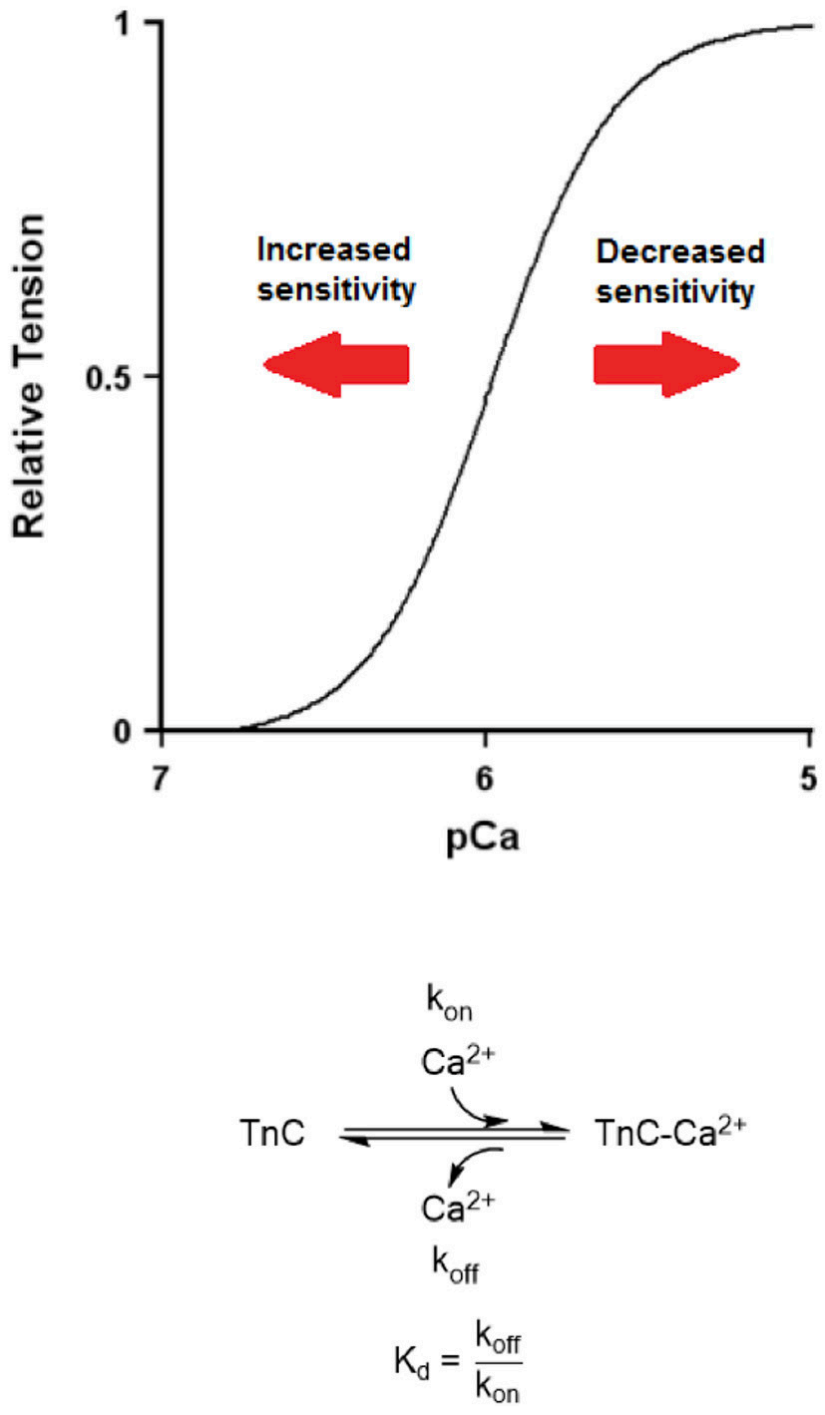
**A hypothetical force-pCa curve demonstrating left- and right-shift (increased and decreased calcium sensitivity, respectively)**. Bottom. A biochemical equation showing calcium association rate to TnC (k_on_) and calcium dissociation rate from TnC (k_off_). A simple equation showing the relationship between equilibrium dissociation constant (K_d_), k_on_, and k_off_.

A typical approach to assess myofilament calcium sensitivity is via construction of a force-pCa curve and determining a potential left- or right-ward shift of the curve (Figure 1). A left-ward shift indicates an increased calcium sensitivity, as a given steady-state force can be attained using a lower concentration of free calcium. On the other hand, a right-ward shift indicates a decreased calcium sensitivity, as a muscle requires a higher concentration of free calcium to generate a given steady-state force. A deeper insight into this steady-state model reveals that, while a change in myofilament calcium sensitivity can reflect altered dynamic behavior, one must also know at least one additional parameter to do so. The equilibrium dissociation constant, K_d_, of TnC is a ratio between the calcium association rate constant to TnC (k_on_) and the calcium dissociation rate constant from TnC (k_off_) (Figure 1). TnC however does not work in isolation (Davis and Tikunova, 2008; Biesiadecki et al., 2014). There are many factors that collaboratively change the sensitivity of the myofilament activation and deactivation by calcium. No current models fully explain the complex integration of all components on the governing of thin-filament calcium binding (see Siddiqui et al., 2016). Thus, for the remainder of this review, we will discuss on on-rate (k_on_) and off rate (k_off_), as the apparent on- and off-rates of the myofilament system, reflecting the effective on- and off-rates of myofilament activation and deactivation not necessarily reflecting solely Ca^2+^ binding to TnC.

**Figure 2:**
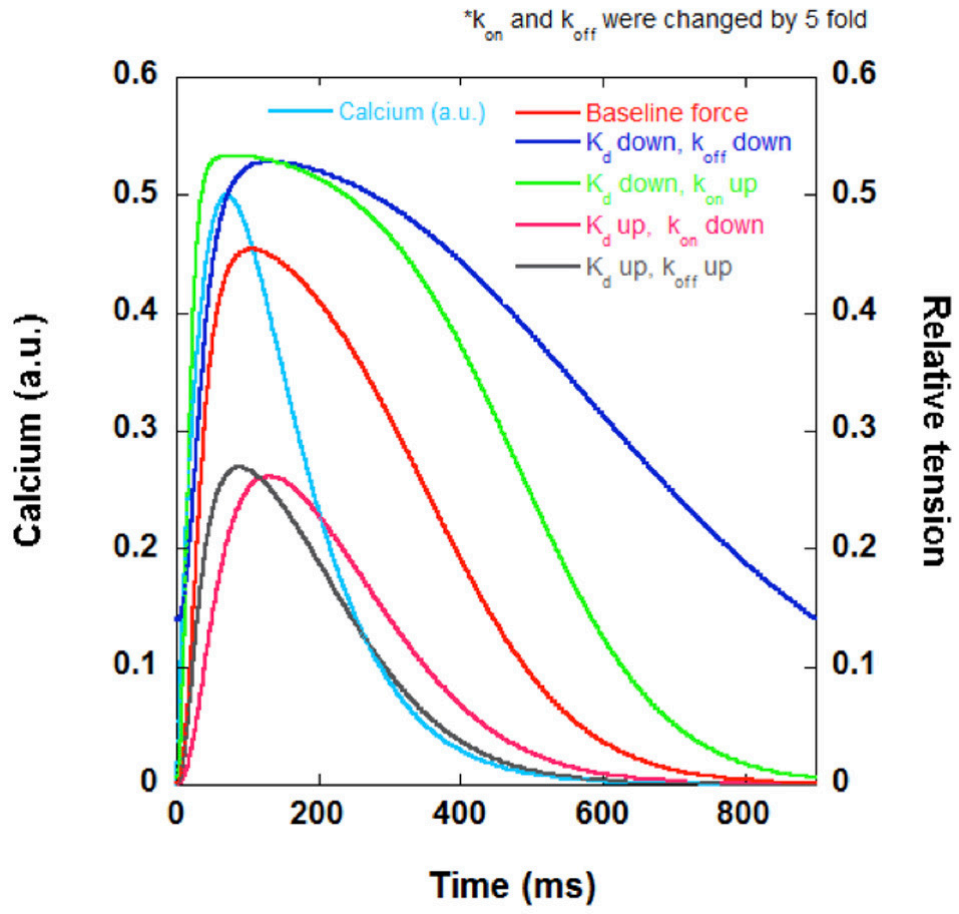
**A set of hypothetical twitches generated using a Labview (National Instruments) program demonstrating the possible effects of altered calcium sensitivity on twitch kinetics**. If the decrease in calcium sensitivity (increase in K_d_) is primarily due to decreased k_on_, one would observe lower developed force (pink). If the decrease in calcium sensitivity is primarily due to increased k_off_, one would observe lower developed force and faster relaxation kinetics (gray). If the increase in calcium sensitivity (decrease in K_d_) is primarily due to decreased k_off_, one would observe increased developed force and slower relaxation kinetics (dark blue). If the increase in calcium sensitivity (increase in K_d_) is primarily due to increased k_on_, one would observe increased developed force and faster relaxation compared to the case where k_off_ is decreased (green). Calcium transient (light blue) and original twitch (red) are also included in the figure.

**Figure 3:**
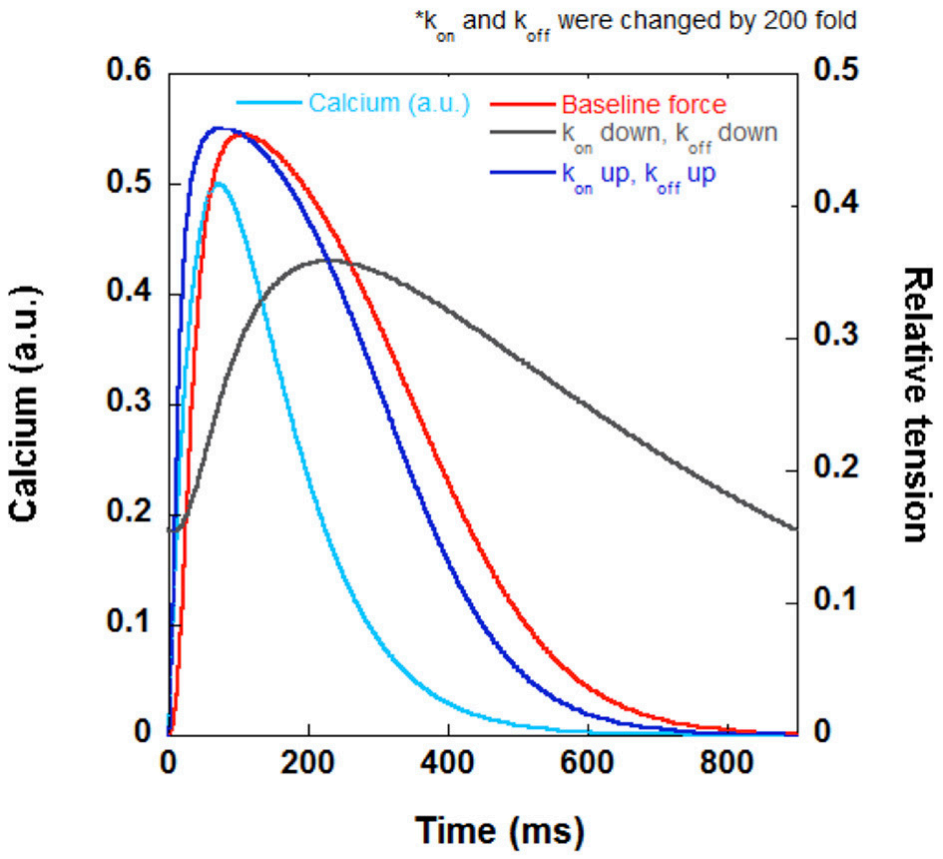
**A set of hypothetical twitches generated using a Labview (National Instruments) program with the same K_d_ demonstrating the effect of modulating k_on_ and k_off_**. When k_on_ and k_off_ are both increased by the same factor to yield the same K_d_, the contraction and relaxation kinetics speed up (dark blue) and begins to more closely resemble the kinetics of the calcium transient (light blue). When k_on_ and k_off_ are both decreased by the same factor to yield the same K_d_, the muscle cannot relax completely at steady-state and has a lower developed force (gray). The original twitch (red) has the same K_d_ as the other two tracings (dark blue and gray).

Myofilament calcium sensitivity increases when the k_on_ increases relative to the k_off_, resulting in an overall decrease in K_d_. In other words, the k_on_ does not necessarily have to increase to increase TnC's calcium sensitivity. As long as the k_off_ decreases by a larger percentage compared to the k_on_, one would observe an increase in calcium sensitivity. This is an important distinction because having an absolute increase in the k_on_ would lead to increased activation of the myofilament and thus increased force generation in our model of cardiac muscle twitch (Figure 2). Our model is written in Labview (National Instruments) and uses a simple mathematical calcium transient: [Ca^2+^]_i_ = Amplitude^*^time^*^e^∧^(-Downamplitude^*^time/τ). This calcium transient (light blue trace in both Figures 2, 3) with kinetic parameters that reflect literature values drives, *via* on and off rate, the thin filament activation level (reflecting TnC-Ca^2+^ binding). This thin filament activation allows cross-bridge formation using the simple 2 state model, governed by an on-rate (f), and an off-rate (g). The model incorporates cross-bridge attachment and detachment rates and thin filament activation levels to generate twitches in real time. Our program allows us to change various parameters such as temperature, calcium transient relaxation constant, cross-bridge attachment rate, and cross-bridge detachment rate. In all the used simulations in this review (Figures 2, 3), all parameters other than the calcium-TnC k_on_ and k_off_ rates were kept constant. Our model reports various twitch kinetic parameters such as time-to-peak (TTP), and relaxation to 50% (RT50) in real time. We generated our cardiac twitches by initially changing these parameters to best mimic the typical cardiac muscle twitch kinetics we have observed in intact human trabeculae (Milani-Nejad et al., 2015). It is believed that the contraction kinetics of a muscle are much slower than the k_on_, which has traditionally been believed to be diffusion-limited, and that changes in the k_on_ do not affect the contraction kinetics (Bers, 2001; Davis and Tikunova, 2008). Therefore, an increase in the k_on_ would result in an increase in developed tension but not necessarily in faster contraction kinetics. On the other hand, having an absolute decrease in the k_off_ would result in slowed relaxation (Figure 3). The rate-limiting steps of relaxation kinetics are complex and involve several distinct processes that at least partially overlap in time. As reviewed in previous literature, the main processes involved are thought to be the decline of the intracellular calcium concentration, transient calcium coming off TnC (k_off_), and cross-bridge cycling kinetics (Biesiadecki et al., 2014). Therefore, a decrease in the k_off_ of TnC could slow down relaxation kinetics. An increase in the k_on_ or a decrease in the k_off_ would culminate in an increased calcium sensitivity but would have drastically different effects on dynamic twitch kinetics.

Despite the unchanged calcium sensitivity, significant consequences for dynamic contraction can occur when the k_on_ and k_off_ increase or decrease by the same factor (Figure 3). This would result in no apparent change in steady-state calcium sensitivity, however, if the k_on_ and k_off_ both increase, the muscle would develop a higher force and relax faster. If the k_on_ and k_off_ both decrease, the muscle would develop a lower force and relax slower. Again, this type of hypothetical analysis reveals that a second important reason that simply measuring the myofilament calcium sensitivity as a stand-alone measurement is not sufficient for translation into dynamic contraction and relaxation.

Another way to determine the dynamic contraction and relaxation behavior of a muscle is by measuring the twitch kinetic parameters of an intact muscle. This information, combined with assessments of force-pCa (intact or skinned), will provide supplemental information on the developed tension as well as the contraction and relaxation kinetics, which can also allow, albeit indirect, more insight into the relative contribution of k_on_ and k_off_, as changes in k_on_ would more heavily influence the developed tension while k_off_ the relaxation kinetics. In summary, the assessment of a force-pCa curve of a muscle at steady-state does not contain sufficient information to make an unambiguous translation into dynamic behavior of a muscle. When dynamic twitch kinetic parameters are not available, but k_on_ and/or k_off_ are assessed in addition to assessment of steady-state myofilament sensitivity, the translation of skinned fiber data toward potential dynamic behavior is greatly enhanced.

To our knowledge, it is virtually impossible to assess the k_on_ and k_off_ in intact muscles, as researchers have typically used *in vitro* fluorimetry to measure changes in fluorescence in isolated TnC at steady-state, but the knowledge of the two rate constants can yield important insights into the contraction and relaxation kinetics of a working muscle (Tikunova and Davis, 2004). Since the dynamic behavior is critically important in various disease states, assessment of kinetics, as well as kinetic reserve is critically important to further understand cardiac malfunction in disease (Janssen et al., 2016).

## Regulation of calcium sensitivity via length, frequency, and beta-adrenergic activation

Cardiac output is heavily regulated, and this regulation is mainly governed by three mechanisms: length-dependent activation, frequency-dependent activation, and beta-adrenergic activation (Janssen, 2010). These three factors all encompass modulation of myofilament calcium sensitivity, which has important implications for the dynamic behavior of cardiac muscle, and we will discuss each of these factors below in more detail.

### Length-dependent activation

The Frank-Starling mechanism is an inherent property of the heart that allows an increase in stroke volume as ventricular volume increases during diastole (Frank, 1895; Knowlton and Starling, 1912). At the level of cardiac muscle, as a muscle is stretched, its developed force per cross-sectional area increases, and this phenomenon is known as length-dependent activation. This effect is beneficial in a cardiac cycle because the ventricular walls are most stretched at the end of diastole, i.e., at the end of the filling phase. The Frank-Starling effect allows the heart to pump the blood to both the lungs and the body with an increased amount of pressure when it is most needed (when the ventricles are more filled with blood). It has been shown by many groups over the past decades that increased muscle length leads to increased overall myofilament calcium sensitivity (Hibberd and Jewell, 1982; Harrison et al., 1988; Dobesh et al., 2002; Herron et al., 2006; Edes et al., 2007). In intact rat and human trabeculae, increased muscle length resulted in increased developed force, as expected, but also results in a slower contraction and relaxation kinetics, exhibiting increased TTP, decreased +dF/dt/F, increased time from peak tension to 50% relaxation (RT50), and decreased −dF/dt/F (Milani-Nejad et al., 2013, 2015). It has been noted that there is no significant increase in intracellular calcium concentration during the fast phase after a stretch, but there is a slow increase in intracellular calcium concentration and developed force during the slow phase, which could account for increased developed tension (Allen and Kurihara, 1982). On the myofilament side, a decrease in lattice spacing (which occurs as a muscle is stretched) has been reported to result in increased calcium sensitivity (Wang and Fuchs, 1995). However, another study has found no increase in calcium sensitivity due to decreased lattice spacing (Konhilas et al., 2002). The length dependence of calcium sensitivity may also involve the number of attached cross-bridges, as it has been noted that the length-dependence of calcium sensitivity disappears when vanadate was used to prevent actin-myosin interaction (Hofmann and Fuchs, 1987). Interestingly, Allen and Kentish noted that calcium sensitivity continues to increase even when a muscle is stretched beyond optimal length, which should result in a decrease in the number of attached cross-bridges (Allen and Kentish, 1985). The phosphorylation status of myofilament proteins such as troponin I or myosin binding protein C (cMyBP-C) can play a role in length-dependent activation (Wijnker et al., 2014; Mamidi et al., 2016). Furthermore, the protein titin is thought to play a significant role in the process of length-dependent activation (Ait-Mou et al., 2016). The overall increase in developed tension and slower relaxation kinetics at greater muscle lengths suggest that the length-dependent increase in calcium sensitivity is probably due to an increase in the k_on_ of calcium as well as a larger decrease in the k_off_.

### Frequency-dependent activation

The frequency of contraction itself has an effect on the amount of developed force in the heart. Increased stimulation frequency results in modification of developed tension, and this is known as the Bowditch effect (Bowditch, 1871). Typically, large animals such as rabbits and humans exhibit a positive force frequency relationship (FFR) (Endoh, 2004). In addition, researchers have noted an accelerated rate of relaxation at increased stimulation, also known as frequency-dependent acceleration of relaxation (FDAR) (Kassiri et al., 2000; DeSantiago et al., 2002). FDAR is required in muscles because the cardiac muscle must return to its relaxed state faster at high heart rates, as it spends less time in diastole. Varian and co-workers have found in intact rabbit trabeculae that increased stimulation frequency leads to FDAR as well as decreased calcium sensitivity (Varian and Janssen, 2007). At least in larger mammals, the decreased calcium sensitivity is accompanied by increased developed tension (due to increased intracellular calcium concentration) and increased rate of relaxation. This suggests that the k_off_ is increased to result in calcium desensitization. Varian et al. has found that troponin I (TnI) and myosin light chain-2 phosphorylation are significantly increased as stimulation frequency is increased from 1 to 4 Hz in intact rabbit trabeculae (Varian and Janssen, 2007). Although Varian et al. did not investigate the phosphorylation of specific amino acid residues in TnI, the phosphorylation status of TnI stimulated at 4 Hz was not significantly different from that stimulated at 1 Hz with isoproterenol, which suggests that the increased TnI phosphorylation at 4 Hz may be primarily due to activation of the protein kinase A pathway (Varian and Janssen, 2007). Serine 23/24 are the most extensively characterized phosphorylation sites of TnI. Phosphorylation of serine 23/24 is known to desensitize the myofilament, which makes these two sites potential phosphorylated sites in the context of increased stimulation frequency (Layland et al., 2005). However, one study on intact rat trabeculae actually found no difference in myosin light chain 2 as well as TnI phosphorylation status at high stimulation frequency (9 Hz) compared to low stimulation frequency (1 Hz) (Lamberts et al., 2007). The effect of increased stimulation frequency on the phosphorylation status of myofilament proteins still remains unclear, and there is ample room for further investigation. The increase in developed tension and faster twitch kinetics at increased stimulation frequencies suggest that the k_on_ and k_off_ are both increased. The overall desensitization of the myofilament at increased stimulation frequencies is likely due to a greater increase in the k_off_ than in the k_on_ to result in overall increase in K_d_.

### Beta-adrenergic activation

When our body is under stress, the adrenal gland releases hormones such as epinephrine and norepinephrine to cope with the stress. One of the effects of these hormones is activation of the beta-adrenergic pathway in the cardiomyocytes, predominantly via the beta1 receptor. This is a useful mechanism for the heart to increase its contractile force, heart rate, and contraction and relaxation kinetics as the demand for oxygen is increased in the body. It is generally accepted in the literature that calcium sensitivity is decreased in response to beta-adrenergic activation (Herzig and Rüegg, 1980; Strang et al., 1994; de Tombe and Stienen, 1995). This is primarily due to the phosphorylation of serine 23/24 in TnI (Layland et al., 2005). Myosin binding protein C is reported to be involved as well, as Cazorla et al. has reported that cMyBP-C knock-out mice had blunted PKA-dependent desensitization (Cazorla et al., 2006). The reduction in calcium sensitivity is accompanied by increased developed force as well as faster contraction and relaxation kinetics, which may suggest that the k_on_ and k_off_ may both increase but the k_off_ has a proportionately larger increase (Zhang et al., 1995; Milani-Nejad et al., 2015). However, the increase in developed force is believed to be predominantly due to an increase in intracellular calcium concentration, rather than the change in myofilament calcium sensitivity (Roof et al., 2011). Robertson et al. have explored the effect of phosphorylation of TnI on the k_on_ and k_off_ and saw that the k_off_ significantly increased upon phosphorylation of TnI but the k_on_ remained the same (Robertson et al., 1982).

## Calcium sensitivity in skinned vs. intact cardiac muscle

Most previous studies that assessed myofilament calcium sensitivity have utilized skinned muscle preparations, reporting approximate half-max force at pCa of 6 (EC_50_). However, intact muscles have been reported to exhibit higher calcium sensitivity compared to skinned muscles (Gao et al., 1994; Varian et al., 2006; Monasky et al., 2010). Later studies, in intact muscle at physiological temperature (Varian et al., 2006; Monasky et al., 2010) confirmed a high sensitivity for calcium in intact muscle compared to published values in skinned/permeabilized muscle. After the muscle skinning process, many intracellular components excluding the myofilament are lost. This naturally leads one to wonder what sensitizing intracellular components are lost during the process of muscle skinning. One possibility might be various kinases that increase the calcium sensitivity of myofilaments. For example, protein kinase D (PKD) has been reported to increase calcium sensitivity via phosphorylation of Ser^315^ cardiac myosin binding protein C (cMyBP-C) (Dirkx et al., 2012). However, PKD can also reduce calcium sensitivity via phosphorylation of troponin I (TnI) Ser^23/24^ (Cuello et al., 2007). It may be possible that there are “natural” calcium sensitizers other than kinases that are (partially) lost upon skinning. Carnosine-like compounds and taurine are examples of cytosolic compounds that have been shown to alter myofilament calcium sensitivity that may be lost during permeabilization (Steele et al., 1990; Lamont and Miller, 1992). Recently, S-glutathionylation of cMyBP-C as well as phosphorylation of TnI by adenosine monophosphate (AMP) kinase have been shown to increase calcium sensitivity (Nixon et al., 2012; Patel et al., 2013). Phosphatase 2A (PP2A), which is associated with various calcium handling and myofilament proteins such as the L-type calcium channel and myosin light chain 2 (MLC-2), also increases calcium sensitivity (Wijnker et al., 2011).

However, it is important to note that there are many desensitizing cytosolic components as well. For example, other kinases such as protein kinase A (PKA) and protein kinase C (PKC) have both been reported to decrease calcium sensitivity, not increase it (Herzig and Rüegg, 1980; Strang et al., 1994; de Tombe and Stienen, 1995; van der Velden et al., 2006). It has been well-documented that protein kinase A (PKA) phosphorylation of TnI 23/24 results in desensitization of myofilament (Layland et al., 2005). Protein kinase C (PKC) and protein kinase G (PKG) can phosphorylate myofilament proteins such as cMyBP-C to reduce calcium sensitivity (Pfitzer et al., 1982; van der Velden et al., 2006). O-linked N-acetyl-D-glucosaminylation of cardiac myofilament also decreases calcium sensitivity (Ramirez-Correa et al., 2008).

Another aspect to consider with the skinning procedure is that sarcomeric lattice spacing increases due to the procedure (Irving et al., 2000). A number of studies have reported that decreased lattice spacing (analogous to increased muscle length) leads to increased calcium sensitivity (McDonald and Moss, 1995; Wang and Fuchs, 1995). The increase in sarcomeric lattice spacing in skinned muscle preparations may lead to decreased calcium sensitivity. However, Konhilas et al. (2002) have reported that osmotic compression of the lattice spacing does not affect the length-calcium sensitivity relationship.

It is not clear at the moment whether desensitizing or sensitizing cytosolic components play a bigger role in intact muscle preparations. In addition, most of the investigations on these cytosolic components were performed on skinned muscle *in vitro*, which makes it difficult for one to predict their actual roles *in vivo*. However, one must carefully consider the implication of the loss of cytosolic signaling molecules and the changes in the myofilament geometry on their direct or indirect effect on calcium sensitivity during the permeabilization process.

## Modification of calcium sensitivity and dynamic behavior of a muscle via myofilament protein mutations

Myofilament proteins work in conjunction to allow the cardiac muscle to contract and relax in response to changes in intracellular free calcium ion concentration. Therefore, it is not surprising that genetic mutations in many of the myofilament proteins impact calcium sensitivity. However, only a few mutations have been characterized to show their translation into dynamic behavior of a muscle. For the purpose of characterization of dynamic contraction of a muscle *in* vivo, it is necessary but not sufficient to show changes in calcium sensitivity. One must also either report biochemical changes in the k_on_ or k_off_ or report twitch force development and kinetics in an intact muscle to show what exact contributing factors changed calcium sensitivity. In this review, we highlight a few mutations that have originally been found in patients with dilated cardiomyopathy (DCM) or hypertrophic cardiomyopathy (HCM) and discuss how these mutations affect myofilament calcium sensitivity and dynamic behavior.

### Troponin C

Troponin C (TnC) is the “calcium sensor” of the myofilament that directly binds calcium at its N-terminus domain to cause a cascade of conformational shifts of myofilament proteins to generate force. TnC L29Q mutation is the first TnC mutation found in a HCM patient, and Liang et al. (2008) have shown that the mutation increases calcium sensitivity in recombinant skinned mouse cardiomyocytes. In addition, the investigators reported an increase in k_on_ but no change in k_off_, which suggests that developed force would be increased in but relaxation kinetics would not be affected in a dynamically contracting muscle (Liang et al., 2008). Interestingly, a later study found no changes in calcium sensitivity in mouse papillary muscles reconstituted with TnC L29Q, and another study actually reported a decrease in calcium sensitivity (Neulen et al., 2009; Gollapudi and Chandra, 2012).

### Troponin I

Troponin I (TnI) is a myofilament protein that inhibits actin and myosin binding by binding to actin in the absence of calcium binding to TnC. Upon binding of calcium to TnC, it releases actin and binds to the hydrophobic patch in the N-terminal domain of TnC to allow interaction between myosin and actin. It has potential implications in the development of HCM, as it has been found in 7% familial HCM (Richard et al., 2003). The TnI R145G HCM mutation increases calcium sensitivity, which was attributed either to increased cross-bridge cycling kinetics or to decreased calcium k_off_ from TnC (Wen et al., 2008). They found that cross-bridge cycling kinetics did not change in the transgenic mice and therefore concluded that decreased k_off_ was the main reason for the increase in calcium sensitivity. Wen et al. also reported reduced maximal force in the TnI R145G mice, which suggests that the k_on_ was probably reduced and at least not increased. This leaves the decreased k_off_ as the main contributor for the increased calcium sensitivity. In addition, the relaxation kinetics were slower in the TnI R145G mice papillary muscle, further supporting the investigators' notion that the k_off_ was decreased (Wen et al., 2008). Since TnI has multiple active phosphorylation sites, cross-talk between different phosphorylation sites adds an additional layer of regulation (also see Salhi et al., 2016).

### Troponin T

Troponin T (TnT) interacts with TnI, TnC, Tm, and actin and therefore can regulate the activity of many myofilament proteins (Gordon et al., 2000). Considering its central position in the troponin complex, it is not surprising that 15% of familial HCM patients exhibit mutations in TnT (Sheng and Jin, 2014). The work by Sommese et al. has revealed that R141W and R173W DCM mutations lead to decreased calcium sensitivity, increased K_*d*_, and increased k_off_ (Sommese et al., 2013). This suggests that the decrease in calcium sensitivity is due to increased k_off_. Deletion of TnT K210, a mutation found in DCM patients, results in desensitization of myofilament in a knock-in mouse model (Du et al., 2007). The investigators also used intact left ventricular papillary muscle to assess its twitch kinetics and reported no change in developed force but faster relaxation (Du et al., 2007). Although the k_on_ and k_off_ were not determined, one can infer from the twitch kinetics data that the decreased calcium sensitivity and faster relaxation kinetics can probably be attributed to the increased k_off_.

### Myosin heavy chain

Myosin heavy chain (MHC) is the force-generating myofilament protein that undergoes power strokes due to its conformational shift. It has two isoforms: α-myosin, the faster isoform, and the β-myosin, the slower isoform. Large mammals such as rabbits and humans express predominantly β-myosin, and small mammals such as mice and rats predominantly express α-myosin (Hoh et al., 1978). Approximately 41% of familial HCM patients have a mutation in the β-myosin heavy chain gene, MYH7 (Richard et al., 2003). A study by Blanchard et al. reported increased calcium sensitivity due to familial HCM mutation R403Q in mouse papillary muscle (Blanchard et al., 1999). However, another study by Palmer et al. did not find any significant changes in calcium sensitivity (Palmer et al., 2008). Chuan et al. measured twitch kinetic parameters at the single cardiomyocyte level and found that the developed force did not change but relaxation kinetics such as RT50 were significantly slower (Chuan et al., 2012). Another study by Kim et al. reported slower contraction and relaxation kinetics in mouse cardiomyocytes, but the cells were unloaded and therefore could not yield information regarding isometric force production (Kim et al., 1999). These results together suggest that the increased calcium sensitivity reported by Blanchard et al. is likely due to decreased k_off_.

### Myosin regulatory light chain

Myosin regulatory light chain (RLC), also known as myosin light chain-2 (MLC-2), is part of the myosin protein that modulates cardiac contraction. Phosphorylation of MLC-2 by myosin light chain kinase (MLCK) is the mechanism via which MLC-2 can affect force development and cross-bridge cycling (Moss and Fitzsimons, 2006). However, mutations in MLC-2 can also influence cardiac contraction and myofilament calcium sensitivity. For example, E22K mutation is one of the first mutations found in familial HCM that culminates in increased calcium sensitivity in skinned glycerinated mouse left ventricular papillary muscle (Szczesna-Cordary et al., 2005). A subsequent study by the same group in freshly skinned mouse left ventricular papillary muscle actually found no significant change in calcium sensitivity due to the MLC-2 E22K mutation (Szczesna-Cordary et al., 2007). This study also used intact papillary muscle and reported decreased force development and faster relaxation kinetics (Szczesna-Cordary et al., 2007). Based on the twitch kinetics data in the 2007 paper, one would predict that k_on_ to decrease and k_off_ to increase to result in an increase in K_d_ and a decrease in calcium sensitivity. However, 2005 and 2007 papers reported either an increase in calcium sensitivity or no change. Further investigation is needed to clearly determine the effect of the E22K on the dynamic twitch kinetics of a cardiac muscle.

## Conclusion

The myofilament is crucial in the regulation of contractile and relaxation behavior of the cardiac muscle, especially in the pathophysiology of heart failure. Force-pCa curves generated from skinned muscle preparations are able to reduce the complex environment of a muscle into a much simpler relationship between isometric force and free calcium ions. While such data are necessary and important, as a stand-alone assessment however, the knowledge of myofilament calcium sensitivity alone is not sufficient for the extrapolation to dynamic behavior of a muscle representative of that *in vivo*. Heart failure continues to be one of the leading causes of death in the U.S., and standard-of-care treatment of the disease has been largely limited to beta blockers, diuretics, angiotensin converting enzyme inhibitors, and calcium channel blockers, which have been in use for decades. More studies on calcium sensitivity that incorporate either assessment of k_on_ or k_off_, or assess dynamic behavior are needed in the field of cardiac physiology to improve interpretation of the impact of myofilament mutations, and for strategizing of novel treatment for the patients who continue to suffer from the disease.

## Author contributions

PJ: concept of review, concept of illustrations, edited final draft. JC: wrote initial draft, made illustrations. MZ, BB, JD: helped discuss concept, edited final draft.

### Conflict of interest statement

The authors declare that the research was conducted in the absence of any commercial or financial relationships that could be construed as a potential conflict of interest.
